# Healthcare professional education in shared decision making in the context of chronic kidney disease: a scoping review

**DOI:** 10.1186/s12882-023-03229-8

**Published:** 2023-06-29

**Authors:** Björn Meijers, Karolien Wellekens, Marco Montomoli, Karmela Altabas, Jessica Geter, Kate McCarthy, Thierry Lobbedez, Rumeyza Kazancioglu, Nicola Thomas

**Affiliations:** 1 Department of Nephrology, Department of Microbiology, Immunology and Transplantation, UZ Leuven, KU Leuven, Leuven, Belgium; 2 Department of Nephrology, Hospital Clínico Universitario, Valencia, Spain; 3 Department of Nephrology and Dialysis, UC Sisters of Mercy, Zagreb, Croatia; 4 Baxter Healthcare Corp, Deerfield, IL USA; 5 Baxter Healthcare Ltd, Compton, UK; 6 Department of Nephrology, University Hospitals of Caen, Caen, France; 7 Department of Nephrology, Bezmialem Vakif University, Istanbul, Türkiye Turkey; 8 London South Bank University, London, UK; 9 Department of Nephrology, University Hospitals Leuven, Herestraat 49, B-3000 Leuven, Belgium

## Abstract

**Rationale & objective:**

Shared decision making (SDM) is a collaborative effort between healthcare professionals, individuals with CKD whereby clinical evidence, expected outcomes and potential side-effects are balanced with individual values and beliefs to provide the best mutually decided treatment option. Meaningful SDM is supported by effective training and education. We aimed to identify the available evidence on SDM training and education of healthcare professionals caring for people with chronic kidney disease. We aimed to identify existing training programs and to explore what means are used to evaluate the quality and effectiveness of these educational efforts.

**Methodology:**

We performed a scoping review to study the effectiveness of training or education about shared decision making of healthcare professionals treating patients with kidney disease. EMBASE, MEDLINE, CINAHL and APA PsycInfo were searched.

**Results:**

After screening of 1190 articles, 24 articles were included for analysis, of which 20 were suitable for quality appraisal. These included 2 systematic reviews, 1 cohort study, 7 qualitative studies, and 10 studies using mixed methods. Study quality was varied with high quality (n = 5), medium quality (n = 12), and low quality (n = 3) studies. The majority of studies (n = 11) explored SDM education for nurses, and physicians (n = 11). Other HCP profiles included social workers (n = 6), dieticians (n = 4), and technicians (n = 2). Topics included education on SDM in withholding of dialysis, modality choice, patient engagement, and end-of-life decisions.

**Limitations:**

We observed significant heterogeneity in study design and varied quality of the data. As the literature search is restricted to evidence published between January 2000 and March 2021, relevant literature outside of this time window has not been taken into account.

**Conclusions:**

Evidence on training and education of SDM for healthcare professionals taking care of patients with CKD is limited. Curricula are not standardized, and educational and training materials do not belong to the public domain. The extent to which interventions have improved the process of shared-decision making is tested mostly by pre-post testing of healthcare professionals, whereas the impact from the patient perspective for the most part remains untested.

**Supplementary Information:**

The online version contains supplementary material available at 10.1186/s12882-023-03229-8.

## Introduction

Individuals and their families living with CKD face a protracted disease course and are confronted with important decisions impacting day-to-day life, e.g., whether or not to start kidney replacement therapy, which dialysis modality to choose, whether or not to enroll in pre-transplantation work-up. In the past, these important decisions were sometimes considered the realm of healthcare professionals. However, most people expect to be involved in the decision-making process, although the preferred level of participation differs between individuals [1].

Shared decision-making (SDM) is a collaborative effort between healthcare professionals, individuals with CKD and their carers/ family whereby clinical evidence, expected outcomes and potential side-effects are balanced with individual values and beliefs to provide the best mutually decided treatment option. An increasing body of evidence supports the use of SDM as it improves decisional quality and satisfaction [2].

Given the relevance of SDM in a wide array of healthcare-related choices, provision of adequate training and education to healthcare professionals (HCP) is needed. Müller et al. performed a systematic review on the quality and effectiveness of healthcare professional training in SDM across different fields of medicine [3]. In sum, the diversity of evaluation methods and the insufficient quality of published evaluations resulted in limited evidence regarding education and training on this topic. To obtain substantial empirical evidence, consensus on validated outcome measures on all stakeholder levels is needed.

In nephrology, a number of practice guidelines advocate the use of SDM. These include the 2000 RPA/ASN guidelines on end-of-life care [4, 5], the European consensus conference in 2015, and the National Institute for Care Excellence (NICE) guidelines on SDM [6]. Over the last two decades, there has been a progressive increase in the uptake of SDM in the care of individuals with CKD. Of note, most of these guidelines do not provide detailed guidance on how best to train and educate teams of healthcare professionals to enable the SDM process.

In this scoping review we aimed to identify the available evidence on SDM training and education of healthcare professionals caring for people with CKD. We aimed to identify existing training programs and to explore what means are used to evaluate the quality and effectiveness of these educational efforts. From this we targeted to identify potential gaps and needs in training and education in SDM for healthcare professionals caring for individuals with CKD.

## Methodology

Due to the diverse range of literature, a scoping review allows for a breadth of concepts and diversity in the study methodologies to be considered. This systematic scoping review methodology examined and mapped qualitative and quantitative evidence in relation to SDM HCP education in kidney care. The PRISM-ScR reporting guidelines for systematic scoping reviews were followed [7]. Studies relevant to SDM education of kidney care HCP were included (supplementary table S1).

### Information sources and search

Databases EMBASE, MEDLINE, CINAHL and APA PsycInfo were searched for relevant studies based on the Population, Exposure and Outcomes of interest (supplementary table S2), with the assistance of an experienced librarian (JG). Full search strings are available in the supplementary materials (supplementary table S3). The databases were searched for the period January 2000 to March 2021 on the 7th and 8th of March 2021.

### Selection of source evidence

Two authors independently reviewed titles and abstracts for inclusion, as per the criteria in supplementary table S1. Any lack of consensus was resolved by majority vote of a third reviewer. Articles selected for full text review were independently reviewed by two authors for eligibility based on in- and exclusion criteria. Lack of consensus during full text review was resolved by discussion until consensus was reached.

### Data extraction and charting process

A formal assessment of study quality was made using the CASP (Critical Appraisal Skills Programme) checklists for systematic reviews, cohort studies, and qualitative studies [8]. The Modified MMAT (Mixed methods appraisal tool) [9] was used for articles having mixed methodologies. Each article was independently assessed by two authors. In case of disagreement of study quality, consensus was reached during discussion.

### Synthesis of results

Data were extracted using data extraction sheets to collect descriptive data such as country/ region of origin, characteristics of healthcare professionals, study design, and outcome measures. The results are reported narratively and in tabular format. Thematic analysis was performed by two authors (KW and BM). The results obtained during initial appraisal were discussed during a group meeting. Additional themes were identified, and the literature was re-analyzed. Synthesis and collation of the extracted information was facilitated by Word 365 and Excel 365.

## Results

### Literature search and article selection

**Fig. 1:**
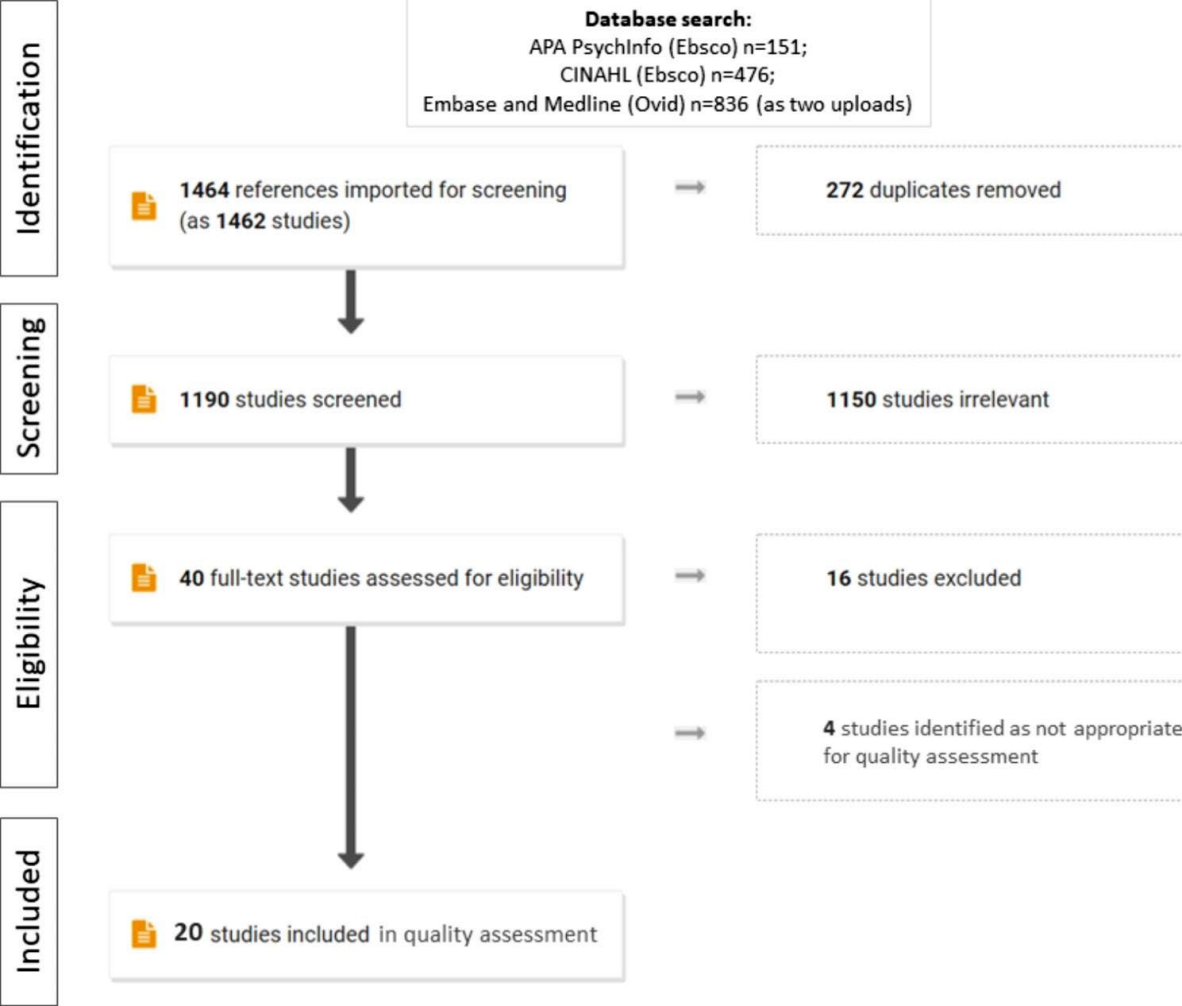
PRISMA-ScR flow diagram

The search strategy, after removal of duplicates, identified 1190 articles for screening (836 from Medline, 0 from EMBASE, 476 from CINAHL and 151 from APA PsycInfo). We excluded 1150 articles based upon abstract review and assessed 40 full text articles based on in- and exclusion criteria. After exclusion of 16 articles, 24 articles have been included in this scoping review (Fig. 1).

20 manuscripts were suitable for quality appraisal. These included 2 systematic reviews, 1 cohort study, 7 qualitative studies, and 10 studies using mixed methods. Study quality was assessed using CASP or MMAT checklists, as appropriate. Study quality was varied and included high quality (n = 5), medium quality (n = 12) and low quality (n = 3) studies as outlined in Table 1. In addition, we identified 4 manuscripts which failed to fit the criteria for quality assessment, including a project proposal, a position statement, a narrative review, and an ethics contribution (Table 1).

**Table 1:** Quality appraisal of included studies

First author	Publication year	Manuscript type	Quality Appraisal tool	Quality score
Hussain et al. [30]	2015	Systematic Review	CASP	High
Murray et al. [31]	2009	Systematic review	CASP	Medium
Singh et al. [32]	2014	Systematic review	CASP	Medium
Ho et al. [11]	2020	Cohort study	CASP	Medium
Davison et al. [33]	2015	Qualitative study	CASP	Medium
Finderup et al. [34]	2019	Qualitative study	CASP	High
Hines et al. [35]	2001	Qualitative study	CASP	Low
Joseph-Williams et al. [20]	2019	Qualitative study	CASP	Medium
Ladin et al. [36]	2018	Qualitative study	CASP	Medium
Rix et al. [37]	2016	Qualitative study	CASP	Medium
Silen et al. [38]	2008	Qualitative study	CASP	Medium
Barnes et al. [17]	2013	Mixed methodology	MMAT	Low
Berzoff et al. [14]	2020	Mixed methodology	MMAT	Low
Combes et al. [39]	2017	Mixed methodology	MMAT	High
Fung et al. [40]	2016	Mixed methodology	MMAT	Medium
Goff et al. [12]	2019	Mixed methodology	MMAT	High
Holley et al. [19]	2007	Mixed methodology	MMAT	High
Luckett et al. [41]	2107	Mixed methodology	MMAT	Medium
Rabetoy et al. [42]	2007	Mixed methodology	MMAT	Medium
Simpson et al. [10]	2019	Mixed methodology	MMAT	Medium
Bordelon et al. [43]	2002	Narrative Review	N/A	N/A
Gordon et al. [44]	2013	Position statement	N/A	N/A
Loiselle et al. [45]	2011	Protocol	N/A	N/A
Rinehart et al. [46]	2013	Ethics	N/A	N/A

CASP, critical appraisal skills programma; MMAT, mixed methods appraisal tool; N/A, not applicable

### Descriptive data

The main characteristics of included studies are summarized in Table 2. Most articles originated from the United States (n = 12, of which n = 3 were not suitable for quality appraisal) and the UK (n = 5). Other countries included Australia (n = 2), Canada (n = 2, of which n = 1 was not suitable for quality appraisal), Denmark (n = 1), Taiwan (n = 1), and Sweden (n = 1).

**Table 2:** Involvement of health care professionals and patients

First author	Publication year	Manuscript type	Country/ region	HCP involvement	Patient involvement
Hussain et al. [30]	2015	Systematic Review	USA	Physicians Nurses Social Workers Dieticians Technicians Administrators	Yes
Murray et al. [31]	2009	Systematic review	Canada	Physicians	Yes
Singh et al. [32]	2014	Systematic review	USA	Physicians	Yes
Ho et al. [11]	2020	Cohort study	Taiwan	Physicians Case Managers	Yes
Davison et al. [33]	2015	Qualitative study	UK	Nurses Administrators	Yes
Finderup et al. [34]	2019	Qualitative study	Denmark	Dialysis coordinators	Yes
Hines et al. [35]	2001	Qualitative study	USA	Nurses	Yes
Joseph-Williams et al. [20]	2019	Qualitative study	UK	Physicians Nurses	Yes
Ladin et al. [36]	2018	Qualitative study	USA	Physicians	No
Rix et al. [37]	2016	Qualitative study	Australia	Nurses	Yes
Silen et al. [38]	2008	Qualitative study	Sweden	Nurses	No
Barnes et al. [17]	2013	Mixed methodology	UK	Nurses	No
Berzoff et al. [14]	2020	Mixed methodology	USA	Social workers	No
Combes et al. [39]	2017	Mixed methodology	UK	Physicians Nurses Social Workers Dieticians Technicians Administrators	Yes
Fung et al. [40]	2016	Mixed methodology	USA	Physicians	No
Goff et al. [12]	2019	Mixed methodology	USA	Physicians Social Workers	No
Holley et al. [19]	2007	Mixed methodology	USA	Physicians	No
Luckett et al. [41]	2107	Mixed methodology	Australia	Physicians Nurses Social workers Psychologists Dieticians Managers	No
Rabetoy et al. [42]	2007	Mixed methodology	USA	Nurses	No
Simpson et al. [10]	2019	Mixed methodology	USA	Nurses Dieticians Social worker	No
Bordelon et al. [43]	2002	Narrative Review	USA	Social Workers	N/A
Gordon et al. [44]	2013	Position statement	USA	Physicians Nurses	N/A
Loiselle et al. [45]	2011	Protocol	Canada	Nurses	N/A
Rinehart et al. [46]	2013	Ethics	USA	Physicians	N/A

This review documents findings in relation to SDM education for kidney healthcare professionals, although a significant number of studies included data obtained from patients. The majority of studies (n = 11) explored SDM education for nurses, and physicians (n = 11). Other HCP profiles included social workers (n = 6), dieticians (n = 4), and technicians (n = 2). Also, the non-study manuscripts were aimed at different healthcare professionals, i.e., nurses, physicians and social workers. Studies mostly explored the pre-dialysis trajectory (commencing/withholding dialysis, n = 11), and end-of-life care (withdrawal of dialysis, n = 8) (Table 3).

**Table 3:** Format of educational interventions

First author	Educational intervention	Topic	Public Availability
Simpson [10]	Lecture	Patient engagement	No
Ho et al. [11]	Lecture Role-playing session	Dialysis commencement and modality choice	No
Goff et al. [12] + Eneanya et al. [13]	Lecture Video Didactic resources	End-of-life	No
Berzoff et al. [14]	Lectures Videos Discussions Case examples Didactic resources	End-of-life	No
Barnes et al. [17]	Learning styles Lectures Patient involvement Role-playing sessions Group discussions	Patient engagement	No
Rinehart [46] + Schell et al. [15] + Schell et al. [16]	Skills training	End-of-life	No

### Types of educational interventions

Some, but not all, articles provide information on the educational intervention(s) (see Table 3). Simpson describes the development of an educational presentation on patient engagement, based on a needs assessment and literature review [10]. The duration of this didactic presentation was 30 min. Ho describes a multi-modal shared decision making program including physician training, development of a decision support tool, telephone interviews and clinical consultations [11]. The training module consisted of a 30-minute introductory presentation followed by a role-playing session. Goff and coworkers describe an intervention study using shared decision making for advance care planning [12]. Detailed information on training for the intervention is given in a separate manuscript [13]. Social workers and nephrologists followed a 60-minute introductory session, and a training tape. The authors provided the URL (uniform resource locator) of this video. However, this video is not publicly available (https://www.youtube.com/watch?v=uzBE7uz3cm4, checked February 27th, 2022). Participants were given didactic resources including a bibliography of recommended literature and the Renal Physicians Association (RPA) guidelines for the initiation and discontinuation of dialysis. Berzoff et al. describe advance care planning training for renal social workers [14]. The curriculum consisted of two parts: a one-day (8 h) didactic training, and longitudinal supervision groups. The didactic training consisted of mixed modality training of themed courses on clinical practice, leadership, culture and spirituality, and legal and ethical issues. Rinehart does not directly report on shared decision-making education, but does refer to a communication skills workshop, called Nephrotalk [15, 16]. Barnes et al. report on a training course to support greater patient engagement in hemodialysis [17]. Two different curricula were developed. A three full-day course, followed by a one full-day course six months later aimed at junior sister/ charge nurses, staff nurses and healthcare assistants. The course uses mixed methodologies, included a learning styles questionnaire, practical motivational interviewing course, patient involvement, as well as theoretical presentations. A shorter one-day course was developed for training of the top tier and was a condensed version of the three-day course.

Loiselle et al. do not report on an actual educational program but provide a protocol for the development of such an intervention. They planned to adapt an existing decision-coaching skill-building workshop [18] to the kidney replacement therapies context. This would include theoretical training on the Ottawa decision support framework, followed by interactive education using pre-recorded videos, role playing and evaluation of interactions. The third and final step of this educational intervention would be practicing with real patients, using self- and peer appraisal. We did not find a follow-up report with the results of this planned development, so have not included this in Table 3.

In addition to an analysis of the educational interventions (the “how”), we also sought to identify the content of educational interventions (the “what”). For this, we explored all articles selected after full-text review, and points of focus in education and training were collected (Table 4). The content can be broadly grouped in two main categories: patient-clinician relationship/ communication and service-related/ organizational factors.

**Table 4:** Content of educational interventions

Patient-clinician relationship/communication	Service-related/organizational factors
Giving an objective overview of options Establishing a trusting relationship Verifying patients prior knowledge together with their desire for information Working to an individualized approach (more ‘counseling’ rather than ‘education’, with regard to time and content, e.g. discussing impact on the daily life) Encouraging patients to participate in the decision (increasing confidence) Training health care staff in emotional support (e.g. leaving room for fear/doubt of patients) Improving skills in bringing bad news Engaging relatives, and learning how to deal with informing relatives Making patients and their relatives reflect (listing pros and cons) Using straight-forward language Summarizing regularly, checking for understanding, and clearly identifying the next steps	Improving clinical expertise for all health care providers, ensuring that informal in-hospital conversations are not biased Creating pre-dialysis opportunities to talk to patients already on dialysis Fine-tuning decision support tools Ensuring private space and taking time for each patient, though considering an accurate workflow Improving cultural and spiritual understanding and support Reflecting in group about ethical dilemmas Routinely establishing prognosis through prediction instruments Providing consecutive appointments, also once patients have started (some patients may think about switching from modality) Offering advanced care planning early in the disease Assisting patients to complete advance care directives Training in palliative support, avoiding therapeutic persistence Obtaining greater institutional engagement Focusing on multidisciplinary cooperation and discussion meetings

### Evaluation of training and education

The second aim of the scoping review was to explore whether and how the quality and effectiveness of these educational efforts were evaluated. We found that several different approaches have been used.

The first approach utilized was to evaluate healthcare professionals’ responses, before and/ or after an educational intervention. Holley et al. investigated whether the RPA/ASN guidelines, published in 2000, have made an impact on health care providers and how they deal with shared-decision making five years later [19]. Based on a questionnaire, assessing how the physician would act in three real-life situations, they concluded that in 2005 there was less variability in withholding dialysis from permanently unconscious patients and in honoring a patient’s Do Not Resuscitate (DNR) request. Goff and coworkers performed qualitative analysis of responses to open-ended questions in surveys [12]. Simpson used a quantitative pretest and posttest design to study “can the educational presentation increase the clinicians’ knowledge about patient engagement” [10]. No statistical difference was found, presumably due to low sample size. Berzoff et al. also aimed at performing a pretest posttest analysis [14]. However, due to significant staff turnover during the intervention period, only about 50% completed both pre-tests and post-tests. To collect additional data, they analyzed supervision calls. Barnes et al. also used a pre and post course questionnaire [17].

A second approach was direct observation of healthcare professional contacts with patients, e.g., during outpatient clinics. Joseph-Williams et al. audio-recorded and transcribed verbatim seventy-six consultations and observed six features of the shared decision making process [20]. In sum, their observation was that real-life shared-decision making did not completely follow the three-talk model. They proposed an alternative descriptive model named ‘implement-SDM’.

A third approach was to evaluate the effects of educational interventions on patients’ experiences. Ho et al. used two scales previously developed by the Ottawa Hospital and Health Research institute (OHHRI) [11]. The decisional conflict scale (DCS) assesses uncertainty in making treatment choices. The decision self-efficacy scale (DSES) probes self-confidence and belief in their abilities for decision making. The studied educational intervention resulted in a significant reduction in decisional conflict, and a significant increase in decisional self-efficacy, with both observed effects persisting for at least one month.

## Discussion

To have a better understanding of how the professionals taking care of people with CKD are trained and educated in shared decision making, we performed a scoping review. We identified 24 studies with diverse methodologies, published between 2007 and 2020. Quality review demonstrated substantial heterogeneity, with review being made more difficult because methods were not reported in a standard format.

Our findings indicated that most studies explored SDM education for nurses and physicians. Types of educational interventions to enhance SDM included lectures, videos, role-play and skills development. The content of educational programs for SDM was broadly grouped in two main categories: patient-clinician relationship/ communication and service-related/ organizational factors. Topics included commencement or withholding of dialysis, modality choice, patient engagement, and end-of-life decisions. We also explored whether and how the quality and effectiveness of these educational efforts were evaluated. We found that several different approaches have been used: healthcare professionals’ responses, before and/ or after an educational intervention; direct observation of healthcare professional contacts with patients; the effects of educational interventions on patients’ experiences.

### Types of educational interventions

Educational programs were reported as taking from 30 min to a 3 full-day program. The educational format comprised either one method, i.e. a theoretical lecture, or skills training, whilst other studies reported on multi-modality training and education, using a combination of theoretical lectures, demo videos, skills labs, and simulation patients. Shorter programs targeted physicians and top tier nursing staff, whereas longer duration multi-modality training and education modules were developed aiming at middle tier nurses and social [21]workers. From the available literature it is not possible to decide what is the most efficacious modality, or optimal duration of the educational intervention. We sought to access the actual training material. None of the studies facilitated public availability, neither as supplement to the publication, nor via referenced online resources. For one intervention an URL was provided [12, 13]. The actual video, however, could not be accessed online. These findings suggest there is much room for improvement. In our opinion, the most effective way in teaching health-care providers in their patient-clinician relationship is by experiential learning, dealing with real-life situations and receiving constructive feedback and reflection afterwards. However, this is an intensive and time-consuming strategy which is therefore not easily applicable. Service-related/organizational factors are more often taught in theoretical classes or online meetings, but hereby the implementation and impact are less easy to verify.

### Evaluation of educational interventions for SDM

The second aim was to identify whether, and if so in what way, educational activities were analyzed for effectiveness. We observed significant heterogeneity. Several articles used a pretest posttest approach probing responses of healthcare professionals [12, 10, 15, 14]. While this approach can capture changes in knowledge and views of professionals, it is not able to capture clinical practice in real-life situations. One study directly observed healthcare professional contacts with patients, capturing audio of these contacts [20]. Although effective, this is very time-consuming. Given normal staff turnover, this is not feasible outside of the experimental setting. Finally, probing the experience of individuals with CKD has been used in one study [11]. This allowed the examination of the effects of educational interventions on decisional quality, using validated scales. The decisional conflict scale (DCS), assessing uncertainty in making treatment choices, and the decision self-efficacy scale (DSES) were able to capture changes in service users’ perceptions in response to a multi-modality educational intervention [11]. In this respect it is noteworthy that the included studies did not make use of validated scores to rate the shared decision making process, e.g. the Observing Patient Involvement scale 5 items or 12 items (OPTION-5/12) [21] [22], or the decision support analysis tool (DSAT-10) [23].

There are several limitations to this scoping review. As the literature search is restricted to evidence published between January 2000 and March 2021, relevant literature outside of this time window has not been taken into account. The current scoping review does not identify all reports on shared decision making in the context of CKD, nor does it aim to. The interested reader is referred to a recent excellent systematic review on this topic [24]. The current review focuses on the process of education and training for healthcare professionals in the context of CKD, including the quality and effectiveness of these educational interventions. We are aware of several impactful programs for shared decision making in the context of CKD, e.g. the OPTION study [25, 26] and ‘My kidneys, My choice’ [27] that were not included in the final selection of this scoping review, as these do not report on evaluation of the quality and effectiveness of educational interventions.

## Conclusions

Despite that healthcare policies, clinical guidelines, and a growing body of evidence [3] favor the widespread implementation of shared decision making, we found limited evidence on how to train and educate healthcare professionals taking care for individuals with CKD. A small number of articles provided information on the curriculum. This showed wide variability in duration, and modalities. None of the reported interventions made the content or training materials publicly available. Evaluation of the effectiveness is mostly done by pre-post testing of healthcare professionals, whereas the impact from the patient perspective for the most part remains untested.

Although broader reviews [28] have highlighted a growing interest in SDM training for HCPs across all clinical practice, it has been found that educational programs are mostly evaluated on a small scale for effectiveness and/or acceptability, and vary greatly in their design, content and delivery. Despite the lack of robust evidence for the impact of education on the quality of shared decision making in chronic kidney disease, we strongly support the recommendations listed below that aim to encourage healthcare teams and educational institutions to include shared decision making throughout their curricula. These recommendations have been supported in other areas of healthcare such as cardiology [29].

**Recommendations**.

1. Education of healthcare professionals in SDM *should* be included in all undergraduate and specialist programs.
2. Educational content on SDM *should* be co-produced with people who have lived experience of CKD, those with educational expertise and also clinicians across the multi-professional team.
3. The content of the educational program on SDM *should* include:

- The evidence for SDM and why it is important for clinicians to effectively communicate benefits and risks with patients.
- Models for SDM (e.g. the Three Talk Model, Elwyn 2017) and how they can be incorporated into clinical practice.
- Key communication skills required for meaningful SDM demonstrated by lived experience examples.
- Ways in which benefits, and risks can be communicated (e.g. the Kidney Failure Risk Equation).
- The potential use of decision support tools and where to locate them.

4. The learning methods of the educational program on SDM *could* include:

- E-learning with local experiential learning time (observation of experienced colleagues).
- Case studies of consultation styles and techniques that enhance shared decision-making.
- Skills labs with simulation and/or recorded observation/feedback.

5. The assessment of the educational program on SDM *could* include:

- Self-test using short answer or MCQs.
- Self-reflection pre- and post-program, with peer review by colleague or patient if appropriate.
- The use of the decisional conflict scale (DCS) or the decision self-efficacy scale (DSES) to capture changes in patients’ perceptions pre- and post-program.

## Electronic supplementary material

Below is the link to the electronic supplementary material.

Supplementary Material 1

## Data Availability

The datasets used and/or analysed during the current study are available from the corresponding author on reasonable request.

## Declarations

**Ethics approval and consent to participate**.

**Consent for publication**.
