# Supplementary material for: Healthcare professional education in shared decision making in the context of chronic kidney disease: a scoping review
Source: BMC Nephrol. 2023 Jun 29;24:195. doi: 10.1186/s12882-023-03229-8 (PMC10308615; doi:10.1186/s12882-023-03229-8)
Supplement: Supplementary file 1 — Supplementary Material 1 [file 12882_2023_3229_MOESM1_ESM.docx]

**Supplementary data**

**Table S1 - Inclusion and Exclusion Criteria**

|  | |
| --- | --- |
| Inclusion Criteria | Exclusion Criteria |
| Studies about the education of healthcare professionals treating people with kidney disease (doctors, nurses, midwives, and allied health) in SDM | Studies about the education of patients only, or evaluation of SDM programmes without reference to the training of HCP treating people with kidney disease. |
| Primary qualitative, quantitative (descriptive and analytic), and mixed methods studies that report kidney care HCP SDM training (process and/or effectiveness) | Studies that are limited to a description of the SDM programme with no description of kidney care HCP SDM training |
| Published in peer reviewed journals | Non-peer-reviewed studies, conference papers, grey literature |
| Published between Jan 2000 and March 2021 | Published before 2000 |
| In English | Not written in English |
| Full text available | No full text available |

**Table S2 – Population, Exposure & Outcome search formulation**

| Population | Health care professionals (HCPs) treating patients with kidney disease. The population concept was addressed with two sets of terms. One for HCPs (including nephrology specialties) and a separate set for patients requiring renal care or dialysis |
| --- | --- |
| Exposure | Training or education about Shared decision making. The exposure concept was addressed with two sets of search terms. One related to HCP education and separate set of terms related to shared decision making. |
| Outcome | Confidence, competence, or effectiveness |

**Table S3 – search strategies and timing**

Ovid search strings: Searched March 7, 2021

**Narrow search:** Covidence Import #1

| 1 | ((Shar* adj3 decision*) or ((participation or participate? or empower* or engag* or involvement or informed or informing) adj7 (patient? or caregiver? or person))).mp. or decision making, shared/ or shared decision making/ or Patient Participation/ or Stakeholder Participation/ | 373779 |
| --- | --- | --- |
| 2 | (Kidney? or renal or CKD or kidney disease or dialysis or peritoneal dialysis or hemodialysis or haemodialysis or kidney transplant* or ESRD or ESKD).mp. or kidney disease/ or chronic kidney failure/ or Kidney Failure, Chronic/ or end stage renal disease/ or dialysis/ or kidney graft/ or Kidney Transplantation/ | 1884690 |
| 3 | (((clinical or profession*) adj competenc*) or (skill* or confidence or satisfact* or attitude* or knowledge or skill? or effective* or evaluation? or prepost or pre test? or post test?)).mp. or Clinical Competence/ | 9793452 |
| 6 | (((nephrologist? or nurs* or physician? or doctor? or healthcare professional? or health care professional? or healthcare provider? or health care provider? or student? or fellow? or pharmacist? or dieti* or counsellor? or social worker? or psychologist? or physiotherapist?).mp. or health care personnel/ or exp medical personnel/ or Health Personnel/) and (educat* or symposium or symposia or train* or survey? or course? or program? or teach* or learn* or intervention? or workshop?).mp.) or Health Personnel/ed or exp medical education/ | 644360 |
| 7 | 1 and 2 and 3 and 6 | 421 |
| 8 | limit 7 to humans | 421 |
| 9 | limit 8 to yr="2000 -Current" | 391 |
| 14 | remove duplicates from 9 | 391 |

**Broader search:** Covidence Import #2

| 2 | (Kidney? or renal or CKD or kidney disease or dialysis or peritoneal dialysis or hemodialysis or haemodialysis or kidney transplant* or ESRD or ESKD).mp. or kidney disease/ or chronic kidney failure/ or Kidney Failure, Chronic/ or end stage renal disease/ or dialysis/ or kidney graft/ or Kidney Transplantation/ | 1884690 |
| --- | --- | --- |
| 3 | (((clinical or profession*) adj competenc*) or (skill* or confidence or satisfact* or attitude* or knowledge or skill? or effective* or evaluation? or prepost or pre test? or post test?)).mp. or Clinical Competence/ | 9793452 |
| 4 | Patient Education as Topic/ use medf or patient education/ use emefd | 165443 |
| 5 | ((Shar* adj3 decision*) or (participation or participate? or empower* or engag* or involvement or informed or informing)).mp. or decision making, shared/ or shared decision making/ or Patient Participation/ or Stakeholder Participation/ | 2446824 |
| 6 | (((nephrologist? or nurs* or physician? or doctor? or healthcare professional? or health care professional? or healthcare provider? or health care provider? or student? or fellow? or pharmacist? or dieti* or counsellor? or social worker? or psychologist? or physiotherapist?).mp. or health care personnel/ or exp medical personnel/ or Health Personnel/) and (educat* or symposium or symposia or train* or survey? or course? or program? or teach* or learn* or intervention? or workshop?).mp.) or Health Personnel/ed or exp medical education/ | 644360 |
| 10 | 2 and 3 and 5 and 6 | 996 |
| 11 | limit 10 to english language | 929 |
| 12 | limit 11 to yr="2000 -Current" | 875 |
| 13 | remove duplicates from 12 | 874 |
| 15 | 13 not 14 | 501 |
| 16 | 15 not 4 | 446 |

PyscInfo: Searched March 8, 2021

Covidence Import #3

| **Search #** | **Terms** | **Results** |
| --- | --- | --- |
| S2 | ( ( nephrologist? OR nurs* OR physician? OR doctor? OR “healthcare professional?” OR “health Search modes - Boolean/Phrase Interface - EBSCOhost Research Databases Search Screen - Advanced 1,098,749 care professional?” OR “healthcare provider?” OR “health care provider?” OR student? OR fellow? OR pharmacist? OR dieti* OR counsellor? OR “social worker?” OR psychologist? OR physiotherapist? ) OR ( (MA “Health Personnel”) OR (DE “Health care services”) ) N7 ((educat* OR symposium OR symposia OR train* OR survey? OR course? OR program? OR teach* OR learn* OR intervention? OR workshop?) OR (DE “Higher education”)) ) OR ( (MA “Medical Education” OR DE "Medical Education" OR DE "Medical Internship" OR DE "Medical Residency" OR DE "Psychiatric Training" OR DE “Nursing education”) ) | 1,098,749 |
| S3 | ( ((Shar* N3 decision*) OR ((participation OR participate? OR empower* OR engag* OR involvement OR informed OR informing) AND (patient? OR caregiver? OR person)) ) OR ( (MA “decision making, shared”) OR (MA “Patient Participation”) OR (MA “Stakeholder Participation” OR DE “Patient centered care” OR DE “Client participation”) ) | 115,874 |
| S5 | Kidney? OR renal OR CKD OR “kidney disease” OR dialysis OR “peritoneal dialysis” OR hemodialysis OR haemodialysis OR “kidney transplant*” OR ESRD OR ESKD OR (MA “kidney Search modes - Boolean/Phrase Interface - EBSCOhost Research Databases Search Screen - Advanced Search Database - APA PsycInfo 12,964 disease”) OR (MA “chronic kidney failure”) OR (MA “Kidney Failure, Chronic”) OR (MA “end stage renal disease”) OR (MA “dialysis”) OR (MA “kidney graft”) OR (MA “Kidney Transplantation”) | 12,964 |
| S6 | ( ((clinical OR profession*) N1 competenc*) ) OR ( (skill* or confidence or satisfact* or attitude* or knowledge or skill? or effective* or evaluation? or prepost or pre test? or post test?) OR (MA “Clinical Competence”) OR (DE “Educational program evaluation” OR DE “Course Evaluation” OR DE “Educational Measurement”) ) | 1,736,911 |
| S7 | (((clinical OR profession*) N1 competenc*) OR ( (skill* or confidence or satisfact* or attitude* or knowledge or skill? or effective* or evaluation? or prepost or pre test? or post test?) OR (MA “Clinical Competence”) OR (DE “Educational program evaluation” OR DE “Course Evaluation” OR DE “Educational Measurement”) )) AND (S2 AND S3 AND S5 AND S6) [Limiters - Publication Year: 2000-; Language: English] | 151 |

CINAHL: Searched March 8, 2021

Covidence Import #4

| **Search #** | **Terms** | **Results** |
| --- | --- | --- |
| S1 | ( ((nephrologist? OR nurs* OR physician? OR doctor? OR “healthcare professional?” OR “health care professional?” OR “healthcare provider?” OR “health care provider?” OR student? OR fellow? OR pharmacist? OR dieti* OR counsellor? OR “social worker?” OR psychologist? OR physiotherapist?) OR (MH "Health Personnel+") ) N7 ((educat* OR symposium OR symposia OR train* OR survey? OR course? OR program? OR teach* OR learn* OR intervention? OR workshop?) OR (MH "Curriculum+") ) ) OR ( (MH "Education, Medical, Continuing") OR (MH "Education, Medical+") OR (MH "Education, Nursing, Continuing") OR (MH "Physicians+/ED") OR (MH "Nurses+/ED") OR (MH "Pharmacists/ED") OR (MH "Pharmacy Technicians/ED") ) | 725,197 |
| S2 | (participation OR participate? OR empower* OR engag* OR involvement OR informed OR Search modes - Boolean/Phrase Interface - EBSCOhost Research Databases 359,549 informing) ) OR (Shar* N3 decision*) OR ( (MH "Decision Making, Shared") OR (MH "Consumer Participation") ) | 359,549 |
| S3 | ( Kidney? OR renal OR CKD OR “kidney disease” OR dialysis OR “peritoneal dialysis” OR hemodialysis OR haemodialysis OR “kidney transplant*” OR ESRD OR ESKD ) OR ( (MH "Hemodialysis+") OR (MH "Kidney Diseases+") OR (MH "Kidney Transplantation") ) | 175,097 |
| S4 | ( (clinical OR profession*) N1 competenc* ) OR ( skill* or confidence or satisfact* or attitude* or knowledge or skill? or effective* or evaluation? or prepost or pre test? or post test? ) OR ( (MH “program evaluation”) OR (MH "Clinical Competence+") OR (MH "Course Evaluation") OR (MH “Educational Measurement”) ) | 2,116,713 |
| S5 | S1 AND S2 AND S3 AND S4 | 476 |
